# QTL on mouse chromosomes 1 and 4 causing sperm-head morphological abnormality and male subfertility

**DOI:** 10.1007/s00335-012-9395-1

**Published:** 2012-03-22

**Authors:** Hideo Gotoh, Keitaro Hirawatari, Naoto Hanzawa, Ikuo Miura, Shigeharu Wakana

**Affiliations:** 1 Agrogenomics Research Center, National Institute of Agrobiological Sciences, 1-2 Owashi, Tsukuba, Ibaraki 305-8634 Japan; 2 Graduate School of Science and Engineering, Yamagata University, 1-4-12 Kojirakawa, Yamagata, 990-8560 Japan; 3 Technology and Development Team for Mouse Phenotype Analysis, RIKEN BioResource Center, 3-1-1 Koyadai, Tsukuba, Ibaraki 305-0074 Japan

## Abstract

The B10.M mouse strain represents a model for male subfertility as it produces a significantly low number of offspring. The only known male reproductive phenotype of this strain is its high frequency of sperm-head morphological abnormalities (44.7 ± 2.4 %). We previously reported that this phenotype was the product of two recessive loci. In this study we mapped the loci causing the high frequency of sperm-head morphological abnormalities in this strain using F2 animals produced by crossing B10.M and C3H mice. Quantitative trait loci (QTL) analysis (*n* = 178) identified two recessive genes, one on Chromosome (Chr) 1 (LOD score = 30.585) and one on Chr 4 (LOD score = 4.532). Further analysis (*n* = 854) mapped the locus on Chr 1 between *Ercc5* (23.55 cM) and *D1Mit528* (25.95 cM) and the locus on Chr 4 between *D4Mit148* (69.48 cM) and *D4Mit170* (70.47 cM). It was also found that the effects of these two loci were not independent. The major locus on Chr 1 determines the expression of sperm-head abnormalities, while the locus on Chr 4 enhances the frequency of abnormalities only when the genotype of the Chr 1 locus is homozygous for the B10.M allele. The major locus on Chr 1 was named sperm-head morphology 1 (*Shm1*), while the modifier locus on Chr 4 was named sperm-head morphology 2 (*Shm2*).

**Electronic supplementary material:**

The online version of this article (doi:10.1007/s00335-012-9395-1) contains supplementary material, which is available to authorized users.

## Introduction

In up to 55 % of subfertile human couples, the male partner is diagnosed with spermatogenic failure (Visser and Repping 2010). Despite the fact that genetic factors are thought to underlie spermatogenic failure, only a few factors have been shown to cause male subfertility (McLachlan et al. 1998; Küpker et al. 1999; Chow and Cheung 2006; Visser and Repping 2010). In domestic animals, although genetic causes for infertility have been assumed, they remain poorly characterized (Steffen 1997). To assess the function of spermatozoa, several tests, e.g., sperm concentration, movement, morphology, cervical mucus penetration, capacitation, zona recognition, the acrosome reaction, sperm–oocyte fusion, oxidative stress, and the integrity of nuclear and mitochondrial DNA, are available (Aitken 2006). A sperm morphology test is one of the requisite test items to evaluate sperm function.

Several studies attempting to locate loci responsible for sperm-head abnormality have been reported. In the genus *Mus*, the phenomenon that hybrid male mice between various mouse species show sterility is well known as hybrid sterility in the mouse (Forejt 1996). L’Hôte et al. (2007) used 24 interspecific recombinant congenic strains of mice between C57BL/6J (*Mus musculus*) strain and SEG/Pas (*Mus spretus*) strain and showed elevated frequencies (up to 47.6 %) of abnormal sperm head compared to the parental strains in them. White et al. (2011) reported a genetic mapping study of hybrid male sterility by quantitative trait loci (QTL) analysis using F2 animals between two inbred strains of mice, PWD/PhJ and WSB/EiJ, which had been raised from two different *Mus musculus* subspecies, *M. m. musculus* and *M. m. domesticus*, respectively. They identified a suite of autosomal and X-linked QTL that underlie measures of hybrid male sterility, including testis weight, sperm density, and sperm morphology.

In the laboratory mice, differences in the frequency of sperm-head abnormalities were found among the strains (Bartke and Krzanowska 1972; Krzanowska 1981), and the genetic factors underlying these abnormalities were investigated (Krzanowska et al. 1995; Golas et al. 2008). We surveyed additional mouse strains (*n* = 17) for the frequency of sperm-head morphological abnormalities and reported strain differences (Gotoh 2010). The B10.M strain showed the highest amount (44.7 ± 2.4 %) of sperm-head abnormalities, and its control strain, C57BL/10J, showed the low amount (4.6 ± 0.6 %). As B10.M is an *h2* congenic strain, whose *h2* region on chromosome (Chr) 17 was introgressed from the stock M mouse at the Jackson Laboratory onto the C57BL/10J strain, a genetic experiment was performed to initially search for the responsible gene in the *h2* region. In this investigation, the contribution of two loci became evident, but these genes were not linked to Chr 17 (Gotoh 2010). Usually, a quantitative phenotype is controlled by multiple genes, and a unique factor of this study was that the frequency of sperm-head abnormalities was quantitative, but the number of controlling genetic factors was shown to be two. In this study, C3H/HeJ was selected as the counterpart of B10.M, and a genetic analysis was performed using their F2 progeny.

## Materials and methods

### Animals

All experimental procedures were approved by our Institutional Animal Care and Use Committee (study identification code #H18-010), and all animals were housed and cared for according to the guidelines established by the Committee. B10.M mice (B10.M-*H2* ^*f*^ *H2-T18* ^*a?*^/SnJ, stock number 000459; The Jackson Laboratory, Bar Harbor, ME, USA) were maintained at our facility for more than ten generations by inbreeding. The name of the subline is B10.M/Sgn. C3H/HeNCrlCrlj mice were purchased from Charles River Japan (Yokohama, Japan). F2 animals were produced by intercrossing the F1 animals obtained from C3H female and B10.M male mice. Animals were maintained on a cycle of 12-h light and 12-h darkness under specific-pathogen-free conditions. A commercial mouse diet (CE-2; Charles River Japan) and water were provided.

### Genotype determination

Polymorphic microsatellite DNA markers that were shared between C3H and C57BL/6J were identified from a public database (RFLP/PCR Polymorphism Query; http://www.informatics.jax.org/searches/polymorphism_form.shtml, Mouse Genome Informatics, The Jackson Laboratory), and 2,971 polymorphic markers were found. We used 159 microsatellite DNA markers for the genome-wide scan (Supplementary Table 1). The microsatellite DNA markers used for the precise mapping of the responsible loci on Chrs 1 and 4 are listed in Table 1. Oligonucleotides were purchased from a commercial supplier (Tsukuba Oligo, Tsukuba, Japan). The methods used for genomic DNA preparation and DNA electrophoresis and the PCR conditions were described previously (Gotoh 2010).

**Table 1:** List of microsatellite markers

	Chromosomal position	
	Genetic (cM)	Physical (bp)
Chr 1		
*Ercc5*	23.55	(44, 204, 692)
*D1Mit236*	23.69	(45, 435, 458)
*D1Mit235*	23.83	(45, 801, 585)
*D1Mit234*	23.93	(45, 931, 099)
*D1Mit528*	25.95	(50, 834, 535)
Chr 4		
*D4Mit148*	69.48	(136, 704, 604)
*D4Mit54*	70.02	(137, 446, 452)
*D4Mit158*	70.02	(137, 502, 609)
*D4Mit170*	70.47	(138, 171, 253)

### Sperm-head morphology test

In this study we modified the previously described method (Gotoh 2010). Two- to three-month-old male mice were used for the collection of sperm samples. Epididymides were dissected out and spermatozoa were transferred into 1 ml phosphate-buffered saline (PBS; pH 7.0). One microliter of sperm suspension was spread on a slide glass, dried in air, fixed with ethanol, and observed under a phase-contrast microscope (Nikon, Tokyo, Japan). Classification of sperm-head morphological abnormality was shown in Supplementary Fig. 1.

### QTL analysis and statistical analysis

QTL analysis was performed using R/qtl software (Broman et al. 2003; Broman and Sen 2009). Comparisons between groups were analyzed by one-way analysis of variance using SPSS 16.0 for Windows (SPSS, Inc., Chicago, IL, USA). A probability of *P* < 0.05 was considered statistically significant.

## Results

### QTL analysis

**Fig. 1:**
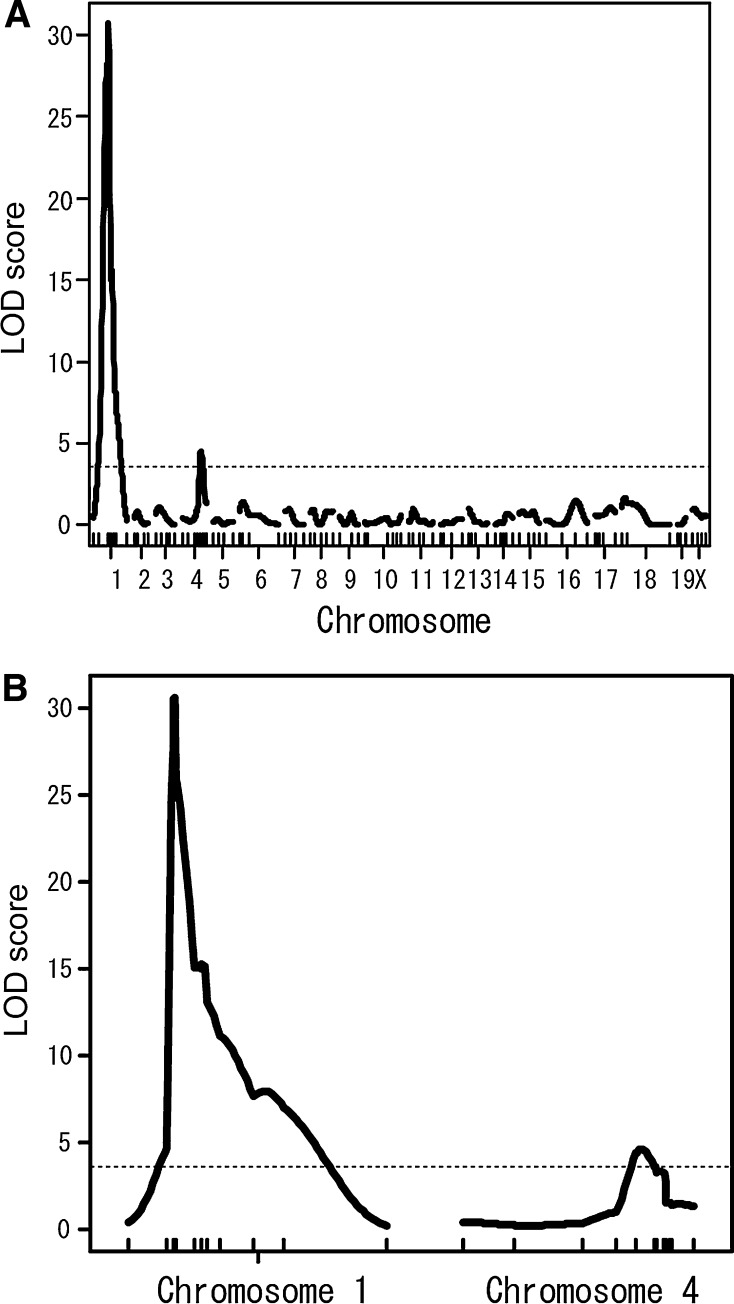
The identification of the two responsible loci on Chrs 1 and 4 by QTL analysis. **a** Whole-genome QTL analysis. Two significant peaks were found on Chrs 1 and 4. The LOD score of the locus on Chr 1 was 30.585 at position 23.7 cM and the LOD score of the locus on Chr 4 was 4.532 at position 70.4 cM. **b** Expanded QTL plot of Chrs 1 and 4. A sharp peak was observed on Chr 1 and a gentle peak was observed on Chr 4. The statistically significant LOD threshold (*P* = 0.05) for the phenotype, as determined by the permutation test (*n* = 1,000), was 3.47 (*dotted horizontal lines*)

QTL analysis was performed on 178 F2 progeny. The results are shown in Fig. 1. Two statistically significant LOD score peaks appeared on Chrs 1 and 4. A sharp peak was found on Chr 1 at 23.7 cM with a LOD score of 30.585. A relatively gentle peak was found on Chr 4 around 70.4 cM with a LOD score of 4.532 (Fig. 1). The locus on Chr 1 was considered to be the major locus for the trait, while the locus on Chr 4 was considered to be the minor locus. We named these loci on Chrs 1 and 4 sperm-head morphology 1 (*Shm1*) and sperm-head morphology 2 (*Shm2*), respectively.

### Inheritance

**Fig. 2:**
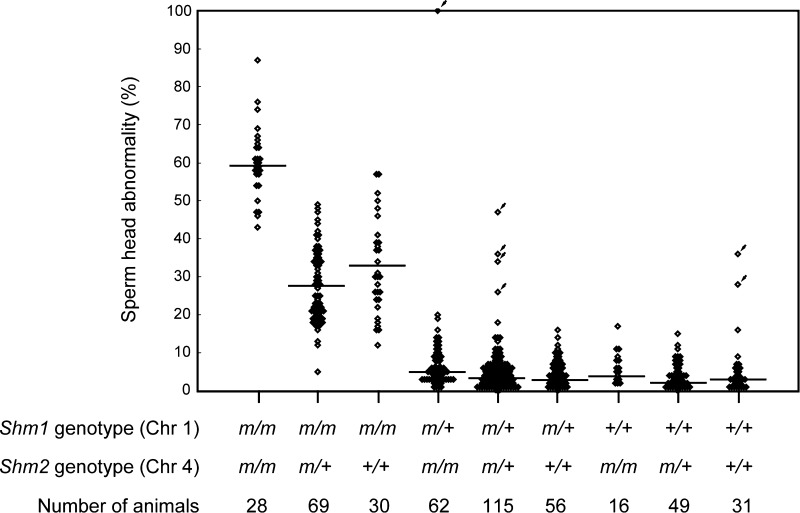
Effects of the *Shm1* locus on Chr 1 and the *Shm2* locus on Chr 4 on sperm-head abnormalities. The frequency of sperm-head abnormalities was plotted for each genotype concerning the responsible loci on Chrs 1 and 4. The genotypes of *D1Mit236* and *D4Mit158* were used for the genotypes of the *Shm1* locus on Chr 1 and the *Shm2* locus on Chr 4, respectively. The alleles from B10.M and C3H were denoted as “*m*” and “*+*,” respectively. Each *square* stands for the value of an individual. The *squares with arrows* indicate that the high frequency of sperm-head abnormalities (>20 %) of these animals was independent of the effects of the B10.M alleles for both of the responsible loci. The *bar* in each genotype represents the mean frequency of sperm-head abnormalities

The frequency of sperm-head abnormalities (*n* = 456) was plotted for each *Shm1* and *Shm2* genotype (Fig. 2). A significant difference was observed between B10.M *Shm1* homozygous mice and mice with other genotypes (*P* < 0.05). A significant difference was also observed within the B10.M *Shm1* homozygotes and between B10.M *Shm2* homozygotes and animals with other genotypes (*P* < 0.05). No significant difference was observed between C3H *Shm2* homozygotes and *Shm2* heterozygotes carrying B10.M *Shm1* homozygously. Among the other genotype groups, including C3H *Shm1* homozygotes and heterozygotes, no significant difference was observed between any genotype combinations.

From the results we concluded that the *Shm1* locus on Chr 1 is the major locus for sperm-head abnormalities, while the *Shm2* locus on Chr 4 modifies the degree of abnormalities positively in a recessive manner only when the genotype of the *Shm1* locus is homozygous for the B10.M allele. Therefore, the *Shm2* locus was considered to be a conditional locus for this trait.

### Genetic mapping

**Fig. 3:**
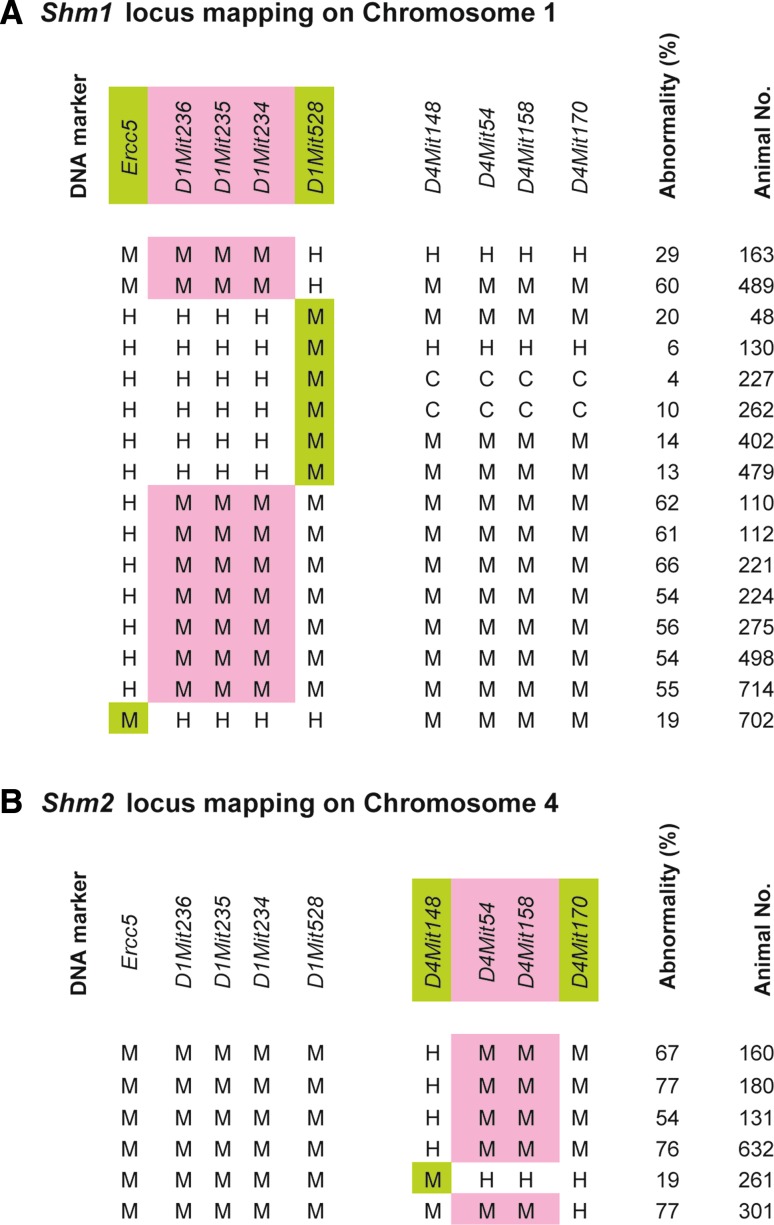
Fine mapping of the responsible loci on Chrs 1 and 4. The genotypes of the informative recombinants whose break points were mapped within the responsible genetic regions on Chrs 1 and 4 are shown. **a** Fine mapping of the *Shm1* locus on Chr 1. **b** Fine mapping of the *Shm2* locus on Chr 4. In order to map the *Shm2* locus on Chr 4, the genotype of the *Shm1* locus on Chr 1 had to be homozygous for the B10.M allele. Homozygotes for the B10.M allele and heterozygotes and homozygotes for the C3H allele are represented as M, H, and C, respectively. The *pink regions* represent the presence of the responsible loci on the B10.M chromosomes. The *light green* chromosomal regions originated from the B10.M chromosomes represent the absence of the responsible loci. The frequency of sperm-head abnormalities and number of animals examined are listed to the right

Genetic mapping analysis was performed according to the genotypes of the F2 progeny (*n* = 854). For mapping *Shm1* on Chr 1, abnormality levels >50% were considered positive for the effect on sperm-head abnormalities when the genotype of *Shm2* on Chr 4 was B10.M homozygous. When the *Shm2* locus was either heterozygous or C3H homozygous, abnormality levels >25% were considered positive, while levels <20% were considered negative. For mapping *Shm2* on Chr 4, the genotype of *Shm1* on Chr 1 had to be B10.M homozygous. Sperm-head abnormality levels >50% were considered positive, while levels <35% were considered negative. The genotypes of the informative recombinants are shown in Fig. 3. Around the peak positions indicated by the QTL analysis, no informative recombination events were obtained between *D1Mit236* and *D1Mit234* on Chr 1 or between *D4Mit54* and *D4Mit158* on Chr 4. The locations of the responsible loci were mapped between *Ercc5* (23.55 cM) and *D1Mit528* (25.95 cM) on Chr 1 and between *D4Mit148* (69.48 cM) and *D4Mit170* (70.47 cM) on Chr 4.

## Discussion

In this study, the positions of the two responsible loci that have positive effects on sperm-head morphological abnormalities in the B10.M strain were clearly defined on Chrs 1 and 4. Currently, *Shm1* on Chr 1 is mapped within a 6.6 × 10^6^-bp region, while *Shm2* on Chr 4 is mapped within a 1.4 × 10^6^-bp region. In both regions, no mutation affecting spermatogenesis has been reported previously.

Based on the establishment of the B10.M line, we suspected cointrogression of the mutant genes from the progenitor stock M population. The stock M mice of the Jackson Laboratory were not inbred mice; however, they are no longer available. Unfortunately, the genetic features of the stock M mice are poorly characterized, although the alleles of the following genes are known: *a* for the agouti (*a*) locus on Chr 2, *Ca* for the keratin 71 (*Krt71*) locus on Chr 15, wild type for the tyrosine-related protein 1 (*Tyrp1*) locus on Chr 4, *b* for the phosphoglycerate kinase 2 (*Pgk2*) locus on Chr 17, *d* for the T region locus 18 of histocompatibility 2 (*H2-T18*) on Chr 17, and *H2* ^*f2*^ for the *H2* haplotype introduced into the B10.M strain on Chr 17 (Snell and Jackson 1958; Flaherty et al. 1977; Eicher et al. 1978; Snell 1978). Genetic typing of microsatellite genes surrounding *Shm1* on Chr 1 and *Shm2* on Chr 4 was uniformly the same as those of the B10 strain, i.e., the control strain for B10.M. Therefore, it was speculated that two independent novel mutations occurred on Chrs 1 and 4 during the establishment of the B10.M strain. Because the mutations are known to affect fertility, it is intriguing to see that the mutations have been maintained. However, it is still possible that a variant gene causing the defect was transferred from the stock M to the B10.M strain. The identification and sequencing of the responsible genes may solve this matter.

Although the confirmed mapping information is convincing, some exceptional cases were observed (Fig. 2, indicated by the arrows). Besides the two genes found in this study, multiple genes affecting the same trait are thought to be present on the genetic background of the progenitor strains. Previously, we found that approximately 1 % of F2 hybrid animals between C3H and B6 showed a wide variety of failure in spermatogenesis (unpublished data). Both parental strains are fertile, and the frequency of sperm-head abnormalities was also normal. The cause of the defects in spermatogenesis was considered to have been caused by the harmful combination of alleles at multiple loci from the two parental strains. Essentially the same phenomena were considered to have occurred in F2 animals from B10.M and C3H crosses. In the mapping study for B10.M mutations, spermatogenic failure that was independent of *Shm1* and *Shm2* was rare. However, careful experimental design will be needed for further analysis. In the next step, we will focus on the fine mapping of the loci and the identification of the genes for this trait.

## Electronic supplementary material

Below is the link to the electronic supplementary material.

Supplementary material 1 (DOC 162 kb)

Supplementary material 2 (TIFF 16495 kb)
