# Supplementary material for: QTL on mouse chromosomes 1 and 4 causing sperm-head morphological abnormality and male subfertility
Source: Mamm Genome. 2012 Mar 22;23(7):399–403. doi: 10.1007/s00335-012-9395-1 (PMC3401295; doi:10.1007/s00335-012-9395-1)
Supplement: Supplementary file 1 — Supplementary material 1 (DOC 162 kb) [file 335_2012_9395_MOESM1_ESM.doc]

|  | **Table S1.** List of microsatellite markers used in this study. | | | | | | | | | | | |  |  |
| --- | --- | --- | --- | --- | --- | --- | --- | --- | --- | --- | --- | --- | --- | --- |
|  |  | **Chr 1** |  |  | *D4Mit44* | 32.65 |  | *D8Mit125* | 21.16 |  | **Chr 14** |  |  |  |
|  |  | *D1Mit64* | 3.67 |  | *D4Mit26* | 42.50 |  | *D8Mit100* | 29.70 |  | *D14Mit11* | 6.33 |  |  |
|  |  | *D1Mit211* | 10.59 |  | *D4Mit332* | 52.02 |  | *D8Mit236* | 40.22 |  | *D14Mit14* | 16.80 |  |  |
|  |  | *D1Mit233* | 20.67 |  | *D4Mit203* | 63.26 |  | *D8Mit211* | 52.00 |  | *D14Mit262* | 28.40 |  |  |
|  |  | *D1Mit213* | 22.88 |  | *D4Mit69* | 68.55 |  | *D8Mit318* | 60.20 |  | *D14Mit7* | 44.98 |  |  |
|  |  | *D1Mit375* | 23.18 |  | *D4Mit251* | 69.05 |  | *D8Mit156* | 76.09 |  | *D14Mit165* | 56.16 |  |  |
|  |  | *Ercc5* | 23.55 |  | *D4Mit68* | 69.44 |  | **Chr 9** |  |  | *D14Mit77* | 64.72 |  |  |
|  |  | *D1Mit236* | 23.69 |  | *D4Mit148* | 69.48 |  | *D9Mit89* | 14.79 |  | **Chr 15** |  |  |  |
|  |  | *D1Mit235* | 23.83 |  | *D4Mit54* | 70.02 |  | *D9Mit328* | 23.53 |  | *D15Mit175* | 3.96 |  |  |
|  |  | *D1Mit234* | 23.93 |  | *D4Mit158* | 70.02 |  | *D9Mit260* | 37.87 |  | *D15Mit138* | 15.68 |  |  |
|  |  | *D1Mit528* | 25.95 |  | *D4Mit170* | 70.47 |  | *D9Mit35* | 51.41 |  | *D15Mit209* | 26.07 |  |  |
|  |  | *D1Mit156* | 33.31 |  | *D4Mit283* | 70.57 |  | *D9Mit116* | 59.58 |  | *D15Mit92* | 32.19 |  |  |
|  |  | *D1Mit303* | 31.79 |  | *D4Mit48* | 73.41 |  | *D9Mit82* | 71.33 |  | *D15Mit171* | 45.02 |  |  |
|  |  | *D1Mit19* | 38.01 |  | *D4Mit284* | 74.64 |  | **Chr 10** |  |  | *D15Mit79* | 58.97 |  |  |
|  |  | *D1Mit7* | 38.55 |  | *D4Mit311* | 75.29 |  | *D10Mit166* | 2.06 |  | **Chr 16** |  |  |  |
|  |  | *D1Mit415* | 43.94 |  | *D4Mit126* | 75.77 |  | *D10Mit213* | 9.75 |  | *D16Mit131* | 3.41 |  |  |
|  |  | *D1Mit187* | 50.84 |  | *D4Mit33* | 81.43 |  | *D10Mit106* | 11.67 |  | *D16Mit165* | 9.66 |  |  |
|  |  | *D1Mit496* | 63.10 |  | **Chr 5** |  |  | *D10Mit3* | 16.53 |  | *D16Mit4* | 25.43 |  |  |
|  |  | *D1Mit107* | 70.19 |  | *D5Mit346* | 2.62 |  | *D10Mit130* | 34.72 |  | *D16Mit139* | 37.28 |  |  |
|  |  | *D1Mit206* | 80.33 |  | *D5Mit61* | 9.98 |  | *D10Mit42* | 39.72 |  | *D16Mit152* | 48.23 |  |  |
|  |  | *D1Mit459* | 91.86 |  | *D5Mit388* | 17.53 |  | *D10Mit12* | 51.15 |  | *D16Mit106* | 57.68 |  |  |
|  |  | *D1Mit155* | 98.20 |  | *D5Mit394* | 29.76 |  | *D10Mit180* | 66.65 |  | **Chr 17** |  |  |  |
|  |  | **Chr 2** |  |  | *D5Mit134* | 38.44 |  | *D10Mit271* | 72.31 |  | *D17Mit164* | 2.11 |  |  |
|  |  | *D2Mit1* | 2.23 |  | *D5Mit41* | 50.68 |  | **Chr 11** |  |  | *D17Mit22* | 17.98 |  |  |
|  |  | *D2Mit295* | 19.97 |  | *D5Mit367* | 60.43 |  | *D11Mit71* | 4.70 |  | *D17Mit152* | 34.90 |  |  |
|  |  | *D2Mit323* | 31.42 |  | *D5Mit163* | 67.99 |  | *D11Mit229* | 15.63 |  | *D17Mit93* | 45.20 |  |  |
|  |  | *D2Mit92* | 42.74 |  | *D5Mit292* | 79.23 |  | *D11Mit242* | 39.47 |  | *D17Mit123* | 60.67 |  |  |
|  |  | *D2Mit15* | 50.66 |  | **Chr 6** |  |  | *D11Mit212* | 54.34 |  | **Chr 18** |  |  |  |
|  |  | *D2Mit304* | 62.49 |  | *D6Mit83* | 5.65 |  | *D11Mit360* | 67.13 |  | *D18Mit164* | 4.65 |  |  |
|  |  | *D2Mit280* | 71.29 |  | *D6Mit268* | 15.12 |  | *D11Mit214* | 80.60 |  | *D18Mit15* | 18.83 |  |  |
|  |  | *D2Mit412* | 82.95 |  | *D6Mit74* | 23.70 |  | **Chr 12** |  |  | *D18Mit58* | 24.58 |  |  |
|  |  | *D2Mit200* | 102.29 |  | *D6Mit188* | 32.53 |  | *D12Mit37* | 2.59 |  | *D18Mit40* | 37.11 |  |  |
|  |  | **Chr 3** |  |  | *D6Mit230* | 45.74 |  | *D12Mit153* | 15.71 |  | *D18Mit7* | 51.92 |  |  |
|  |  | *D3Mit164* | 2.01 |  | *D6Mit10* | 52.75 |  | *D12Mit114* | 28.94 |  | **Chr 19** |  |  |  |
|  |  | *D3Mit151* | 15.18 |  | *D6Mit220* | 64.70 |  | *D12Mit14* | 37.00 |  | *D19Mit16* | 13.82 |  |  |
|  |  | *D3Mit224* | 20.54 |  | *D6Mit15* | 77.70 |  | *D12Mit101* | 51.55 |  | *D19Mit18* | 26.00 |  |  |
|  |  | *D3Mit137* | 34.91 |  | **Chr 7** |  |  | *D12Mit263* | 62.11 |  | *D19Mit83* | 40.41 |  |  |
|  |  | *D3Mit78* | 48.81 |  | *D7Mit191* | 9.38 |  | **Chr 13** |  |  | *D19Mit1* | 51.92 |  |  |
|  |  | *D3Mit14* | 61.32 |  | *D7Mit270* | 25.61 |  | *D13Mit17* | 7.73 |  | **Chr X** |  |  |  |
|  |  | *D3Mit18* | 72.36 |  | *D7Mit346* | 33.88 |  | *D13Mit266* | 14.44 |  | *DXMit54* | 6.64 |  |  |
|  |  | *D3Mit19* | 87.60 |  | *D7Mit31* | 49.01 |  | *D13Mit244* | 21.63 |  | *DXMit166* | 28.26 |  |  |
|  |  | **Chr 4** |  |  | *D7Mit7* | 63.44 |  | *D13Mit66* | 34.54 |  | *DXMit16* | 37.00 |  |  |
|  |  | *D4Mit101* | 4.29 |  | *D7Mit105* | 70.29 |  | *D13Mit126* | 45.05 |  | *DXMit130* | 55.45 |  |  |
|  |  | *D4Mit193* | 13.99 |  | **Chr 8** |  |  | *D13Mit148* | 59.69 |  | *DXMit186* | 76.75 |  |  |
|  |  | *D4Mit89* | 24.66 |  | *D8Mit124* | 7.59 |  | *D13Mit35* | 67.21 |  |  |  |  |  |
|  |  |  |  |  |  |  |  |  |  |  |  |  |  |  |
|  | Names of microsatellite markers and their chromosomal positions from the centromere (cM) are listed (Genome Coordinates, NCBI Build 37). | | | | | | | | | | | | |  |
|  |  |
