# Supplementary figures and images for: QTL on mouse chromosomes 1 and 4 causing sperm-head morphological abnormality and male subfertility
Source: Mamm Genome. 2012 Mar 22;23(7):399–403. doi: 10.1007/s00335-012-9395-1 (PMC3401295; doi:10.1007/s00335-012-9395-1)

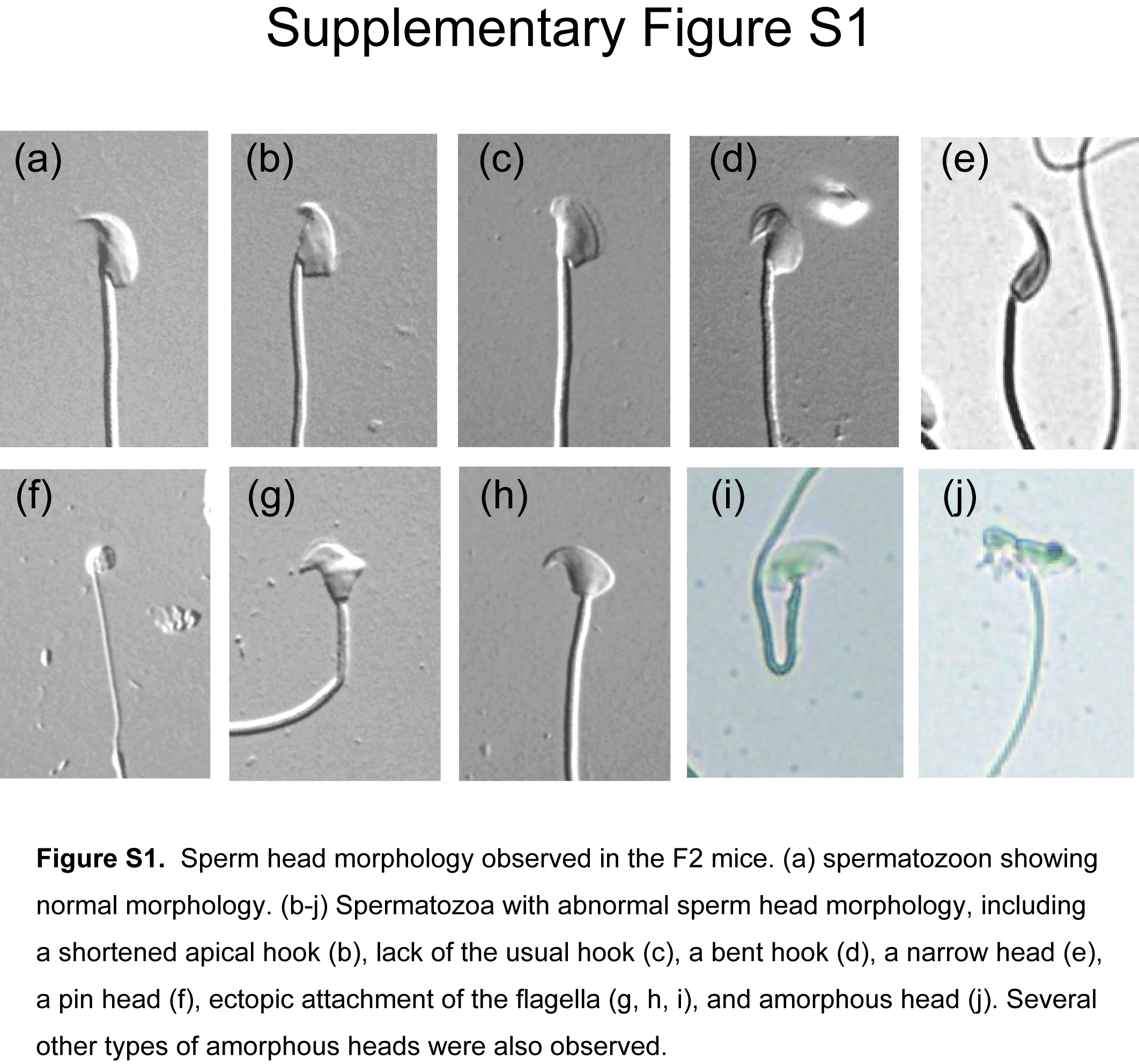

Supplement: Supplementary file 2 — Supplementary material 2 (TIFF 16495 kb) [file 335_2012_9395_MOESM2_ESM.tif]
